# Assessing the Prevalence and Length of the Anterior Loop of Inferior Alveolar Nerve and the Position and Dimension of Mental Foramen Using Cone Beam Computed Tomography

**DOI:** 10.7759/cureus.68535

**Published:** 2024-09-03

**Authors:** Sunita Srivastava, Kavleen K Sethi, Abhishek Sinha, Amitabh Srivastava, Anuj Mishra, Nazish Khan, Shivangi Pandey, Adarsh Dixit

**Affiliations:** 1 Oral Medicine and Radiology, Sardar Patel Post Graduate Institute of Dental and Medical Sciences, Lucknow, IND; 2 Oral Medicine and Radiology, Sardar Patel Post Graduate Institute of Dental and Medical Science, Lucknow, IND; 3 Implantology and Periodontics, Sardar Patel Post Graduate Institute of Dental and Medical Sciences, Lucknow, IND

**Keywords:** position and dimension, mental foramen, cone beam computed tomography, anterior loop of inferior alveolar nerve, dental implant

## Abstract

Background

To prevent harm to the neurovascular bundle during clinical and surgical operations, it is crucial to understand the position and dimension of the mental foramen, as well as the prevalence and length of the anterior loop (AL).

Methods

An iCAT Vision (CT Dent, London, UK) was used to take a cone beam computed tomography (CBCT) scan. Measurement of height, length of anterior loop, position and width of mental foramen was examined.

Results

Assessed prevalence of anterior loop and saw difference among genders, which was found to be more among males than females; anterior loop decreased as age advances. Mean anterior loop length of study subjects in different age group on right side was more than the left side, and the most common location of the mental foramen (61.87% in females and 61.36% in males) is located below the apex of the second premolar. The mean value of the distance from inferior border was 9.72mm in females and 10.78mm in males.

Conclusion

The current study was done with all of these characteristics in mind to assess the effectiveness of CBCT in determining anterior looping of the inferior alveolar nerve (IAN) and the position and dimension of mental foramen. In more than half of the cases analyzed, an anterior loop was discovered.

## Introduction

Dental implants are routinely used in oral rehabilitation. The frequent placement of dental implants has led to an increase in neurosensory disturbances and hemorrhages, even in the anterior mandible, an area previously thought to be safe due to its lack of essential neurovascular bundles [1]. Prior to implant placement, it is crucial to obtain the necessary knowledge regarding the mandible's important structures [2]. Careful and detailed planning is required before commencing implant surgery to identify vital mandibular structures, as well as the shapes and dimensions of bone, to ensure proper orientation and placement of the implants [3].

Many imaging modalities have been recommended for implant treatment planning. However, traditional radiographs often fail to effectively visualize buccolingual width, angulation, or the location of mandibular vital structures [3]. According to the literature, major variations have been reported in the prevalence and length of the anterior loop, and its prevalence is reported to range from 7.7% to 95.2%. Ethnicity, sex, and age differences may exist, making it essential to assess the prevalence and length of the anterior loop and determine a precise and safe distance from it in different populations for clinical significance during surgical procedures [4]. One of the most challenging regions for implants is the mental foramen region. This is because there are many variations in the size and location of the mental foramen. The position of mental foramen varies in the vertical and horizontal planes. When planning for an implant, it is crucial to identify the vertical position of the mental foramen, as after extraction and resorption, the foramen moves closer to the alveolar crest. In adults, the mental foramen is nearer to the inferior border, while it moves upwards closer to the alveolar border in old age due to the loss of teeth and bone resorption. The exact location of the mental foramen is important for both diagnostic and clinical purposes.

The aim of this study was to assess the prevalence and length of the anterior loop of the inferior alveolar nerve and the position and dimension of the mental foramen. The objective was to assess the anterior loop length and to compare with gender age groups between the right and left sides.

## Materials and methods

The study was a prospective study performed in the Oral Medicine and Radiology Department after getting approval from the IEC(FP-01/OMR/2022/IEC) and was in accordance with the Helsinki Declaration of 1975 as revised in 2013. The data was taken from the patient's pool who had visited for some other reasons. Inclusion criteria included patients with bilateral images of the lower cortical boundary at least 2 cm distal to the mental foramen and anterior mandible. Patients 15- 80 years of age of both sexes with dentulous and partially edentulous arches were considered. Pathological conditions that can affect the position of the mandibular canal and mental foramen such as cysts and tumors, fracture lines, supernumerary teeth or unerupted teeth, processing and exposure errors, and artifacts obscuring visibility were excluded from the study. 250 patients, met the necessary inclusion criteria and were enrolled as study subjects.

Methodology

An iCAT Vision was used to take a CBCT scan. All patients were scanned with the following settings: field of view (FOV) measuring 16 x11 cm with a voxel size of 0.2mm, tube voltage of 120kvp, tube current of 20.27 mA, and exposure time of 14.7s. The data pool for this was carried out from November 2021 to February 2022. The images of subjects (who had undergone CBCT examination showing the mandibular premolar/molar region) who had undergone a procedure for some other reasons and inspected for the presence of nerve loop.

**Figure 1:**
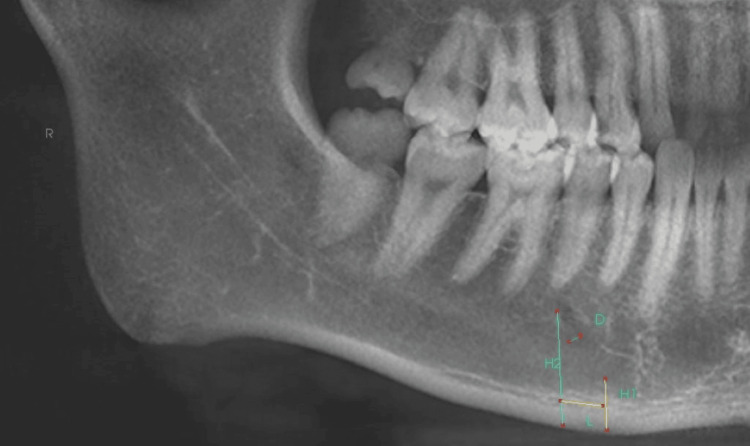
Panoramic section showing different variables of anterior loop assessed in the study

To examine CBCT images single examiner studied and evaluated the images. Measurement of height, length of anterior loop, position, and width of mental foramen was examined. CBCT images and Invivo 5 Anatomage software were used. Multiplanar reconstructions were made from picture sequences and anterior loop presence or absence was recorded once the loop was detected; the length of the anterior loop was calculated by utilizing a number of consecutive coronal reconstructions between the anterior loop border and anterior mental foramen border. This value is multiplied by the slices' thickness (0.3 mm). Cropped Panoramic image showing different parameters of anterior loop measured (H1, H2, L, D) along premolar region (Figure 1)

Measurement of anterior loop length

L stands for the anterior loop length; H1 stands for height from the superior cortex of mental foramen to the lower border of the mandible in panoramic reconstructed images and sagittal sections; H2 stands for height from anteriormost point of AL to the lower border of the mandible in panoramic reconstructed images and sagittal sections; and D stands for anterior loop's diameter

According to Apostolakis and Brown, [5] the anterior loop can be differentiated from the incisive canal based on the fact that the incisive canal has a diameter of <3 mm. When only a single round hypodense image was visualized, it was interpreted as an incisive canal if it exhibited a diameter smaller than 3 mm. If the diameter was larger than 3 mm, the anterior extension of the mandibular canal was considered to be an anterior loop. An anterior loop was also considered to be present when two round hypodense areas were observed, with one corresponding to the lumen of the mandibular canal that traverses the mental foramen anteriorly and inferiorly and the other reflecting doubling back (loop) of the mandibular canal, leading to externalization of Inferior alveolar nerve.

Mental foramen

**Figure 2:**
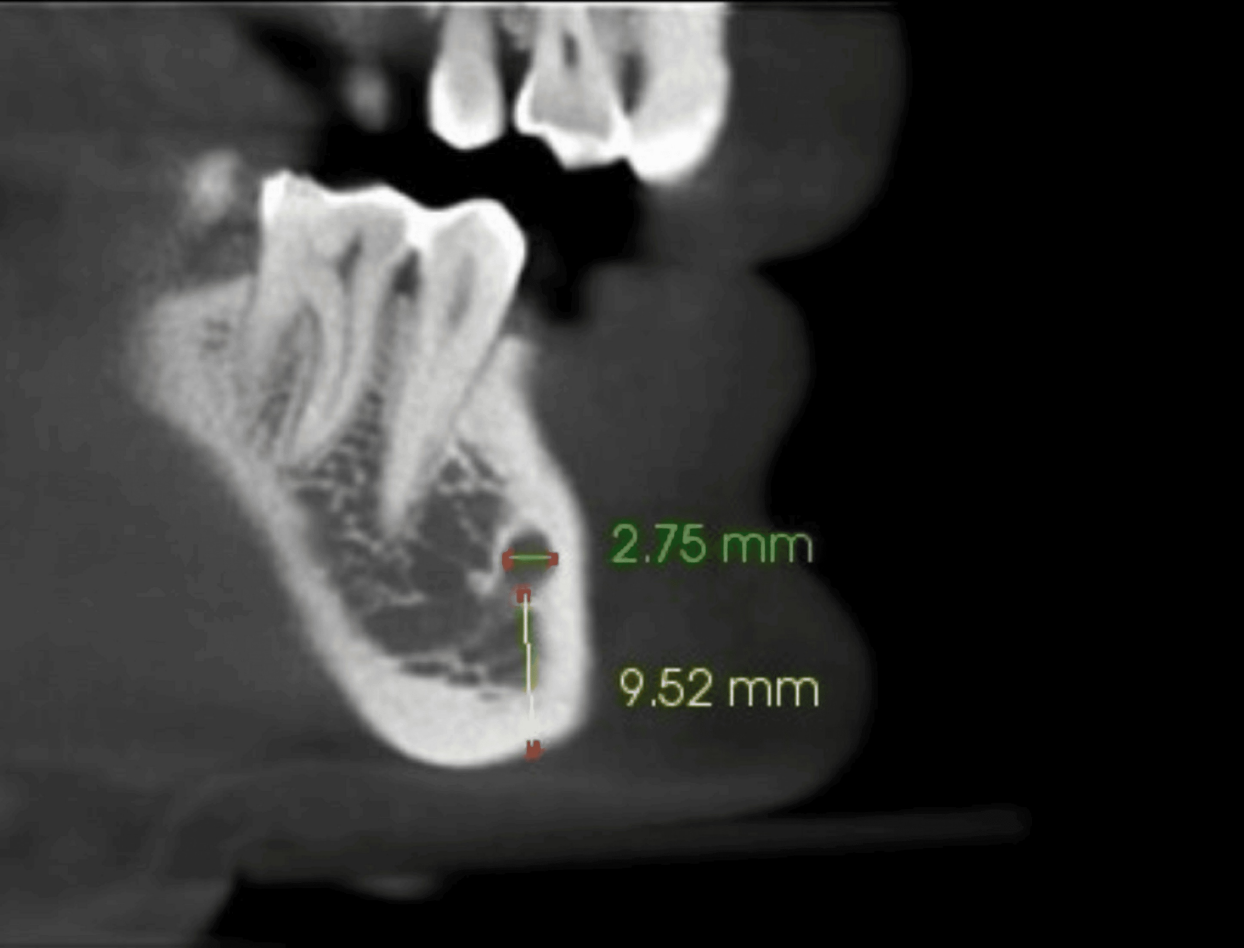
Width and distance of the mental foramen from the inferior border of the mandible

Saggital image showing measurement of dimension and position of mental foramen from the inferior border of the mandible (Figure 2)

The position of the mental foramen was classified in relation to the teeth of the lower jaw in accordance with Tebo and Telford [6]. 

1) Foramen lying on a longitudinal axis passing between canine and first premolar; 2) Foramen lying on the longitudinal axis of first premolar; 3) Foramen lying on a longitudinal axis passing between first and second premolars; 4) Foramen lying on the longitudinal axis of the second premolar; 5) Foramen lying on a longitudinal axis passing between the second premolar and first molar; and 6) Foramen lying on the longitudinal axis of the first molar.

Statistical analysis

Based on a 95% confidence interval and 4% type I error and response distribution of 50 percent, the sample size was estimated to be 482, and taking a 5% attrition rate, the sample size was increased to 500 i.e 250 patients

Intergroup comparison was made using unpaired/independent t-test and one Way ANOVA for difference in mean scores between independent groups. Frequencies between the different groups were compared using the Chi-squared test. Shapiro-Wilk test was used to investigate the distribution of data and Levene's test to explore the homogeneity of Variables.

## Results

To determine the prevalence of looping in each age category, patients, based on their age, were divided into three groups: (i) 15-35 years with 55 (31.61%) subjects; (ii) 36-56 years with 64 (34.41%) subjects; and (iii) >56 years with 52 (37.14%) subjects. The prevalence of anterior looping was 43.19% (114 subjects) in males and 24.16% (57 subjects) in females, with a statistically significant p-value of 0.001 (Table 1).

**Table 1:** Prevalence of loop in relation to gender

	Absent	Present	p-value
Female	179	57	0.001 (Sig)
	75.84%	24.16%	
Male	150	114	
	56.81%	43.19%	

On the right side, the mean values for height from the mental foramen's superior-most cortex to the mandible's lower border (H1) were estimated to be 11.60 mm in males and 10.01 mm in females. On the left side, H1 was assessed to be 10.60 mm in males and 9.50 mm in females. The intergroup comparison between males and females was statistically significant for both the right and left sides (Table 2).

**Table 2:** Comparison of variables of the anterior loop between genders

	Gender	Mean	Std. deviation	Std. error mean	p-value	Significance
Height I-right	Female	10.0185	1.34312	.23381	0.001	Non-significant
	Male	11.6005	1.36168	.18361		
Height II-right	Female	13.3276	1.32082	.22993	0.001	Non-significant
	Male	14.5831	1.37115	.18489		
Height I-left	Female	9.5038	1.97900	.43185	0.001	Significant
	Male	10.6031	1.73155	.24736		
Height II-left	Female	12.4738	1.74856	.38157	0.001	Significant
	Male	14.4257	1.84363	.26338		

For the height from the anterior-most point of the anterior loop to the lower border of the mandible (H2), the mean values on the right side were 14.58 mm in males and 13.32 mm in females. On the left side, the mean values were 14.42 mm in males and 12.47 mm in females. The intergroup comparison between males and females was statistically significant for both sides (Table 2).

The mean anterior loop length in the age group 15-35 years was the maximum, i.e., 7.5 mm on the right side, followed by 36-55 years (6.0 mm). However, the minimum loop length was noted in the age group >56 years as 5.8 mm, with a significant difference seen with respect to age groups. On the left side, the length was similar, with a maximum for 15-35 years (7.4 mm) and the least in the >56 years age group (5.7 mm), which was also statistically significant (Table 3).

**Table 3:** Length of the anterior loop according to age group

		Mean	Std. deviation	Std. error mean	p-value	Significance
Length-right	15-35 years	7.5284	2.59526	.51905	0.010	Significant
	36-55 years	6.0605	1.96922	.31533		
	56-80 years	5.8896	1.59422	.32542		
Length-left	15-35 years	7.4148	1.80610	.36122	0.004	Significant
	36-55 years	6.4748	2.02969	.44292		
	56-80 years	5.7554	1.17327	.23949		

Position of mental foramen

Among females, 61.87% had the mental foramen located below the apex of the second premolar; 27.54% had it between the first and second premolars; and 10.17% had it between the second premolar and the first molar. Among males, 61.36% had the mental foramen located below the apex of the second premolar; 26.89% had it between the first and second premolars; and 9.47% had it between the second premolar and the first molar (non-significant among gender; Table 4).

**Table 4:** Position of the mental foramen in relation to gender PM - premolar

	1st premolar	Between 1st premolar and 2nd PM	2nd Premolar	Between 2nd PM and 1st molar	1st molar	p-value
Female	1	65	146	24	0	0.621 (NS)
	0.42	27.54	61.87	10.17	0	
Male	4	71	162	25	2	
	1.52	26.89	61.36	9.47	0.76	

Size and distance from the inferior border of mental foramen

On the right side, the mean width of the mental foramen was 2.7 mm in females and 2.7 mm in males. On the left side, it was 2.6 mm in females and 2.7 mm in males with no significant difference (Table 5) On the right side, the mean distance of the mental foramen from the inferior border of the mandible was 9.7 mm in females and 10.7 mm in males. On the left side, it was 9.5 mm in females and 11.0 mm in males, with no significant difference (Table 5).

**Table 5:** Width and distance of mental foramen in relation to gender

		Female	Male	p-value	Significance
Width	Right side	2.791±0.944	2.720±0.846	0.529	Non-significant
	Left side	2.615±0.853	2.711±1.156	0.46	Non-significant
Distance	Right side	9.725±1.328	10.785±2.017	0.001	Significant
	Left side	9.588±1.359	11.020±1.691	0.001	Significant

## Discussion

Utilizing a diagnostic modality that enables proper viewing and measurement of the anterior loop is crucial due to the complicated anatomy of the loop. CBCT provides a 3D assessment without distortion, magnification, or unsharpness, as seen in panoramic radiography [6]. The anterior loop contains mental and incisive nerves; care should be taken during surgeries in the interforaminal region to prevent nerve damage [4]. Our study showed a higher prevalence of the anterior loop among males than females, similar to the findings of Uchida et al. [7]. Our study showed a trend where the visibility of the anterior loop decreased with increasing age, demonstrating a prevalence of the anterior loop in a decreasing order with age. These findings were similar to those of a study performed by Katyayani et al., where the prevalence of the anterior loop decreased as age advanced. However, studies done by Apostolakis and Brown [4] found no significant relationships between these variables (age, gender, and side) and the length of the anterior loop.

According to the results of a systematic review, the anterior loop was seen in 40.6% of the population, comprising 34.5% of females and 34.9% of males [8]. This anatomical landmark was observed by Parnia et al. in 84% of their cases, which is much higher than our findings. A wide range is seen for the occurrence of the anterior loop, with the prevalence varying from 49% to 83% [9]. The frequencies of identifying the anterior loop in the studies by Arzouman et al. and Kuzmanovic et al. were 12% and 27%, respectively [9].

Various studies reported mean lengths ranging from 0.4 mm to 6 mm, coinciding with values in literature by Neiva et al. [10], who reported the most extended anterior loop in the literature in the American population to be 11 mm, followed by Uchida et al. [7], in the Japanese population, with an anterior loop length of 9 mm. Our most extended anterior loop measured 7.5 mm in the population. Moghaddam et al. and Antoinette et al. found the mean anterior loop length to be 2.77 mm and 7.25 mm, respectively [11]. Wong et al. studied the Malaysian population and found a mean loop length of 3.85 mm on the right side and 3.69 mm on the left side [12].

Al-Mahalawy et al. [13] found the mental foramen (MF) below the mandibular second premolar in 52.8% of subjects and between the premolars in 29.6%. Similarly, our study found the MF below the second premolar in 61.3% of males and 61.8% of females and between the premolars in 27% of males and 26% of females. Aldosimani et al. [14] reported the MF near the second premolar in over 68.1% of subjects in the Saudi population. Both our study and Al-Mahalawy et al. found no gender differences in MF position, unlike Al-Khateeb et al. [15], who reported it more frequently apical to the second premolar in females and between the first and second premolars in males.

On the right side, the mean distance of the mental foramen from the inferior border of the mandible was 9.7 mm in females and 10.7 mm in males. On the left side, the distance of the mental foramen from the inferior mandibular border was 9.5 mm in females and 11.0 mm in males. There was a significant difference between genders, and according to von Arx et al. [16], the average distance was 13.2 mm and 12.4 mm, respectively. In our study, the width of the mental foramen was 2.7 mm in males and 2.6 mm in females. No significant variation was detected between genders. According to a study by Shalash et al. [17], the width of the MF was 3.41 mm in females and 3.59 mm in males. There was a significant difference between genders regarding the distance of the mental foramen from the inferior border of the mandible.

Limitations

Further studies with larger samples are needed to assess the variability of the anterior loop in order to make a strong recommendation with regard to safety margins.

## Conclusions

Many earlier studies have evaluated the prevalence of the anterior loop but did not see variations with regard to age, gender, and right or left sides. Our study assessed the prevalence of the anterior loop and saw differences among genders, which was found to be more common among males than females; the anterior loop decreased as the age advanced. The mean anterior loop length of study subjects in different age groups on the right side was more than the left side, and mental foramen were typically located below the apex of the mandibular second premolar.
